# Distribution and functions of γδ T cells infiltrated in the ovarian cancer microenvironment

**DOI:** 10.1186/s12967-019-1897-0

**Published:** 2019-05-07

**Authors:** Xian Chen, Wenwen Shang, Rui Xu, Ming Wu, Xiaojie Zhang, Peijun Huang, Fang Wang, Shiyang Pan

**Affiliations:** 1 Department of Laboratory Medicine, The First Affiliated Hospital of Nanjing Medical University, No. 300 of Guangzhou Road, Nanjing, 210029 China; 2 National Key Clinical Department of Laboratory Medicine, No. 300 of Guangzhou Road, Nanjing, 210029 China

**Keywords:** Ovarian cancer, Γδ T cell, Vδ1 T cell, Cytotoxicity capacity, Immunosuppressive activity

## Abstract

**Background:**

The role of γδ T cells, innate-like lymphocytes with unrestrained MHC, in various malignancies has recently been extensively studied. However, there is limited research regarding γδ T cells in ovarian cancer (OC) patients.

**Methods:**

Here, we investigated the distribution patterns of γδ T cells and their main subsets in peripheral blood and tumor tissues among OC patients, benign ovarian tumor (BOT) patients, and age-matched healthy controls (HC) by flow cytometry, as well as the expression levels of IFN-γ and IL-17A secreted from γδ T cells. Immunohistochemical staining was utilized to detect the numbers of γδ T cells and their main subsets in different types of ovarian tumor tissues. Additionally, we also investigated chemotaxis effects on γδ T cells, as well as their cytotoxic activity and proliferation.

**Results:**

We found that the percentages of γδ T cells and Vδ1 T cells were significantly higher in OC tissues than BOT tissues and normal (N) ovarian tissues, while there were no obvious differences in peripheral blood. Meanwhile, higher numbers of γδ T cells and Vδ1 T cells were observed in OC tissues, and were positively related to advanced clinicopathological features of OC patients. Further, the levels of IFN-γ secreted by γδ T cells were relatively lower, while IL-17A was expressed at a high level in both the peripheral blood and tissues of OC patients. Chemotaxis assay revealed that supernatants derived from OC tissues possessed a stronger capacity to attract and recruit γδ T cells. However, γδ T cells sorted from OC tissues showed weakened cytotoxic activity against ovarian cancer cells, and γδ T cells cocultured with OC tissue supernatants could effectively inhibit the proliferative activity of naïve CD4^+^ T cells.

**Conclusions:**

These data suggested that γδ T cells might have critical roles in OC progression and potential utilization in treatment approaches or prognosis prediction.

**Electronic supplementary material:**

The online version of this article (10.1186/s12967-019-1897-0) contains supplementary material, which is available to authorized users.

## Background

Ovarian cancer (OC) is a devastating disease because more than 70% patients are in its advanced clinical stages (III/IV) when first diagnosed. Furthermore, more than 70% of patients suffer relapse and the 5-year survival rate is lower than 40% [1, 2]. Immunotherapy is a promising treatment for numerous solid tumors, including OC [3]. However, the capacity of tumor cells to establish an immunosuppressive microenvironment to evade immune surveillance is the major obstacle in tumor immunotherapy [4, 5]. Therefore, a better understanding of the roles that the immunosuppressive microenvironment has in OC is critical.

Immunosuppressive cells and inhibitory factors are important components of the tumor immunosuppressive microenvironment, and include regulatory T cells (Tregs), marrow-derived suppressive cells (MDSCs), tumor-associated macrophages (TAMs), interleukin-10 (IL-10), and transforming growth factor (TGF-β). Tregs are vital immunosuppressive cells that contribute to the suppression of immune responses, mediate immune tolerance, and participate in the tumorigenesis, development and metastasis of cancer. However, although traditional Tregs including CD4^+^ Tregs and CD8^+^ Tregs, both αβT cell types, have been extensively studied [6], much less is known about γδ T cells in tumor immunity [7].

γδ T cells are major MHC-unrestricted T lymphocytes that are widely distributed in peripheral blood (PB) and mucosal tissues with limited proportions. There are two main subsets, Vδ1 γδ T (Vδ1 T cells) and Vδ2 γδ T (Vδ2 T cells), based on the heterogeneity of the δ chain. Although the essential roles of γδ T cells as a natural element of innate immunity include reactions to tissue stress or damage, malignancy, or microbial infections, the features of γδ T cells could be heterogeneous with regards to impaired immune responses, because they exhibit inhibitory effects at tumor sites [8]. Recently, γδ T cells infiltrated in colorectal, pancreatic, and breast cancer were demonstrated to have multiple actions in promoting tumorigenesis and metastasis [9, 10]. Thus, these discoveries prompted us to investigate the functions of γδ T cells in human OC.

In this study, we demonstrated higher accumulation of γδ T cells and Vδ1 T cells in OC tissues regardless of the relative percentages and numbers, along with partly impaired cytotoxic capacity against OC cells and enhanced immunosuppressive function on naïve CD4^+^ T cells, prompting abetter understanding of γδ T cells in immune suppression and tumor progression of OC.

## Methods

### Patients and specimens

This research was authorized by the Ethical Committee of the First Affiliated Hospital of Nanjing Medical University (Nanjing, China), and written inform consent was obtained from all patients. Fresh tumor tissues and PB samples were obtained from OC and BOT patients treated at the First Affiliated Hospital of Nanjing Medical University from 2016 to 2017. Paraffin-embedded specimens of OC tissues, borderline ovarian tumor (BOT) tissues, and BOT tissues were collected from patients treated at the Nanjing Drum Tower Hospital. None of these patients had received radiotherapy or chemotherapy before specimen collection. PB samples from healthy donors who underwent a physical examination served as healthy controls. The clinical data of the patients were also collected for analysis.

### Separation of mononuclear cells

Peripheral blood mononuclear cells (PBMCs) were obtained from fresh PB using Ficoll-Hypaque (TBD, Tianjin, China) density gradient centrifugation. Fresh OC, BOT, and N tissues were minced into small pieces and then digested in serum-free RPMI 1640 medium containing DNase I (50 U/ml), hyaluronidase (100 μg/ml), collagenase type IV (1 mg/ml), and l-glutamine (2 μM) (all from Sigma, St Louis MO, USA) for 2 h at room temperature with low rotation. Single-cell suspensions were obtained by filtering the digested tissues through 40 μm cell strainers (BD Falcon, San Jose, CA, USA), and tumor-infiltrating lymphocytes (TILs) were isolated by centrifugation of a single-cell suspension on a Percoll gradient (GE HealthCare Life Sciences, Piscataway, NJ, USA).

### Flow cytometry analysis

Freshly isolated PBMCs or TILs (1 × 10^6^) were washed and incubated with fluorophore-conjugated monoclonal antibodies including Alexa Fluor 750-anti-CD45, Alexa Fluor 700-anti-CD3, BV421-anti-TCRγδ, FITC-anti-Vδ1, and PE-anti-Vδ2 (all from Biolegend, San Jose, CA, USA) for 20 min at room temperature in the dark. For intracellular cytokine detection, PBMCs or TILs (1 × 10^6^) were resuspended in RPMI 1640 medium supplemented with 10% FBS (Gibco) and stimulated with phorbol-12-myristate 13-acetate (50 ng/ml), ionomycin (1 μg/ml), and brefeldin (1 μg/ml) (all from Biogems, Rocky Hill, NJ, USA) for 5 h in 5% CO_2_ atmosphere at 37 °C. Cells were then washed, fixed, permeabilized, and stained with APC-anti-IL-17A, and APC/cy7-anti-IFN-γ (Biolegend, San Jose, CA, USA) according to the manufacturer’s protocol. Fluorescence data were collected on a FACS Aria II (BD Biosciences, San Jose, CA, USA) and analyzed with FlowJo software (Tree Star, Ashland, OR, US).

### Immunohistochemistry

The numbers of γδ T cells and Vδ1 T cells infiltrated in OC, OBT, and BOT tissues were detected by immunohistochemistry (IHC). Formalin-fixed and paraffin-embedded tissues were cut into 4-μm-thick sections and deparaffinized, and then rehydrated in xylene and graded ethanol. Then the sections were heated at 100 °C for 3 min in citrate solution (pH 6.0) for antigen retrieval, after which endogenous peroxidase activity was blocked by incubation with 3% H_2_O_2_ for 15 min and heterogenetic antigen was blocked with normal goat serum at room temperature for 2 h. Anti-TCRγδ and anti-TCRVδ1 antibody (1:100 dilution, Beckman Coulter, Genetex, USA) were added and incubated for 18 h at 4 °C. After washing, the sections were incubated with HRP-labeled goat-anti-mouse IgG antibody at 37 °C for 20 min, using diaminobenzidine solution for color development, and were counter-stained with hematoxylin. Negative controls were treated in the same manner except that the primary antibody was omitted. The infiltrated numbers of γδ T cells and Vδ1 T cells in the tissues were assessed manually using a BX51 microscope (Olympus, Japan). Positive cells in the tumor sections was quantitated by counting and the average numbers of positive cells per reported field were calculated in random areas of 10 cancer nests and cancer stroma area using high-power magnification (×400).

### Cell sorting and cell line culture

γδ T cells were sorted from PBMCs or TILs by fluorescence-activated cell sorting (FACS), following staining with Alexa Fluor 750-anti-CD45, Alexa Fluor 700-anti-CD3, andBV421-anti-TCRγδ (all from Biolegend, San Jose, CA, USA), using a FACS Aria II cell sorter; the purity of the sorted cells were greater than 95%.

The human OC cell line SKOV3 was purchased from ATCC (American Type Culture Collection, Manassas, VA, USA), and cultured in McCoy’s 5A medium (Invitrogen, Carlsbad, CA, USA) supplemented with 10% FBS at 37 °C in 5% CO_2_.

### Chemotaxis assay

Tissues supernatants were obtained by centrifugal and filtration culture supernatant from which single cells (1 × 10^6^) from digested OC and BOT tissues were cultured with RPMI 1640 medium containing 10% FBS for 48 h at 37 °C. Chemotaxis assay were performed using 24-wellplates with inner wells (5 μm pore size; Corning Costar, Corning, NY, USA). OC tissues supernatants, BOT tissues supernatants, and RPMI 1640 control medium were added to the outer wells of 24-well plates. γδ T cells (1 × 10^5^) sorted from PB and OC tissues were added to the inner wells containing the three groups. After 150 min incubation, the numbers of γδ T cells migrated to the outer wells were quantified as the results of chemotaxis. The chemotaxis index was calculated by counting the numbers of γδ T cells that had migrated in response to tissues supernatants compared with the responses to control medium alone.

### Co-culture experiments

γδ T cells (1 × 10^5^) sorted from healthy PB were cultured with OC tissues supernatants, BOT tissues supernatants, and RPMI 1640 control medium in 24-well plates in 37 °C at 5% CO_2_. After 5 days, γδ T cells were harvested and stained with Alexa Fluor 750-anti-CD45, Alexa Fluor 700-anti-CD3, BV421-anti-TCRγδ, FITC-anti-Vδ1, and PE-anti-Vδ2 (all from Biolegend, San Jose, CA, USA) to analysis the levels of Vδ1 T cells and Vδ2 T cells by flow cytometry.

### Cytotoxicity assay

γδ T cells originating from PB and OC tissues were incubated with OC cell line SKOV3 at an effect (γδ T cells): target (SKOV3 cells) (E:T) ratio of 10:1 in 96-well plates at 37 °C in 5% CO_2_ in which SKOV3 were fluorescence-labeled by cell trace dye CFSE kit (5 mM, Invitrogen). After 4 h incubation, 7-AAD (1 µg/µl; BD Bioscience) was added to the culture for another 15 min to mark dead cells, and CFSE^+^ 7-AAD^+^ cells were analyzed by flow cytometry as dead SKOV3 cells. The specific lysis of target cells was counted as follows: %(CT − TE)/(1 − CT) × 100%, in which CT was defined as the mean number of fluorescent target cells in control tubes without effector cells and TE indicated the mean number of fluorescent target cells cultured with effector cells.

### Proliferation assay of naïve CD4^+^ T cells

γδ T cells sorted from PB were cultured with OC tissue supernatants or RPMI 1640 control medium for 5 days. Naïve CD4^+^ T cells were negatively selected from healthy donors using a naïve CD4^+^ T Cell Isolation Kit II (Miltenyi Biotec, Germany). γδ T cells (1 × 10^5^) collected from the two groups were co-cultured with naïve CD4^+^ T cells at a ratio of 1:1 in U-bottomed 96-well plates containing anti-CD3 and anti-CD28 antibody (1 μg/ml; eBioscience, San Diego, CA, USA). PBMCs (5 × 10^4^) irradiated with 40Gy were added to the culture as antigen-presenting cells (APCs). After 56 h of culture, [^3^H] thymidine was added at a final concentration of 1 μCi/well and cultured for an additional 16 h. The incorporation of [^3^H] thymidine was measured with a liquid scintillation counter.

### Statistical analysis

Data were analyzed with SPSS (Statistical Package for the Social Science) 20.0 software (IBM Corp, Armonk, NY, USA) and are expressed as mean ± standard error (SEM). Comparison with groups was calculated using Student’s *t*-test or non-parametric Mann–Whitney *U*-test as appropriate. *P* values less than 0.05 were considered statistically significant.

## Results

### Distribution characteristics of γδ T cells in peripheral blood of ovarian cancer

**Fig. 1:**
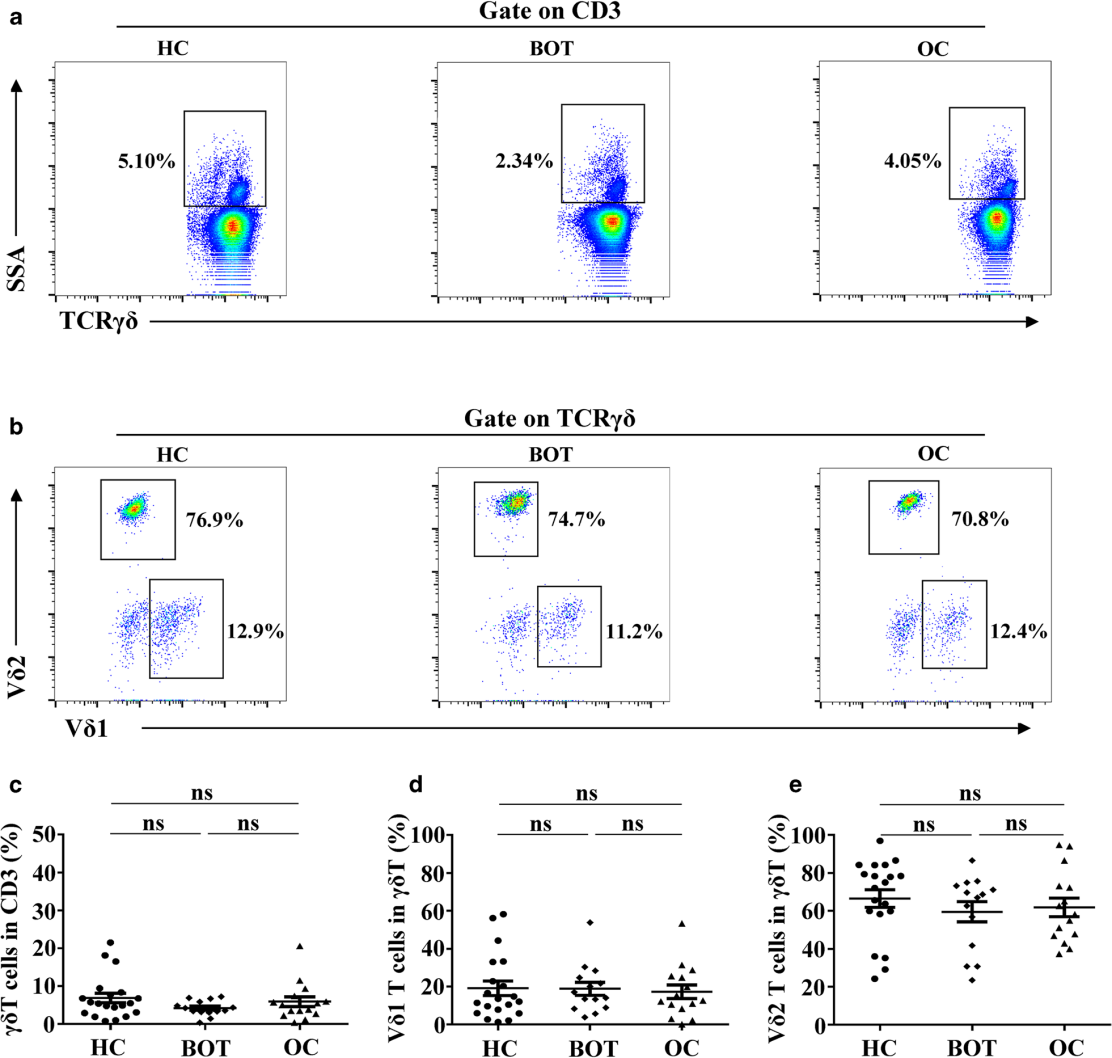
Distribution characteristics of γδ T cells in peripheral blood of ovarian cancer. **a**, **b** Representative dot pots of γδ T cells, Vδ1 T cells and Vδ2 T cells in peripheral blood of HC, BOT patients and OC patients. **c**–**e** Detailed statistical graphs of γδ T cells, Vδ1 T cells and Vδ2 T cells in HC (n = 20), BOT patients (n = 14) and OC patients (n = 15). Data are shown as mean ± SEM

To identify the distribution characteristics of γδ T cells in PB, we measured the relative percentages of γδ T cells in OC patients, BOT patients, and HCs via flow cytometry. As shown in Fig. 1, we found that γδ T cells from CD3^+^ T cells in OC patients showed no obvious differences compared with BOT patients and HCs (6.87% ± 5.68% vs. 4.17% ± 2.01% vs. 5.90% ± 5.03%, *P *> 0.05; Fig. 1a, c). γδ T cells can be divided into two structural subsets, Vδ1 T cells and Vδ2 T cells, due to the heterogeneity of the δ chain. We identified that Vδ1 T cells were evident in less significant percentages inOC patients, BOT patients, and HCs (19.12% ± 17.2% vs. 18.8% ± 12.96% vs. 17.3% ± 13.8, *P *> 0.05; Fig. 1b, d), and comprised less than 20% of all γδ T cells in PB. However, Vδ2 T cells showed no significant difference in OC patients, BOT patients, and HCs (66.4% ± 20.7% vs. 59.5% ± 19.7 vs. 61.8% ± 18.9%, *P *> 0.05; Fig. 1b, e), but was the principal subset of γδ T cells in PB (> 60%).

### Distribution patterns of γδ T cells in tissues of ovarian cancer

**Fig. 2:**
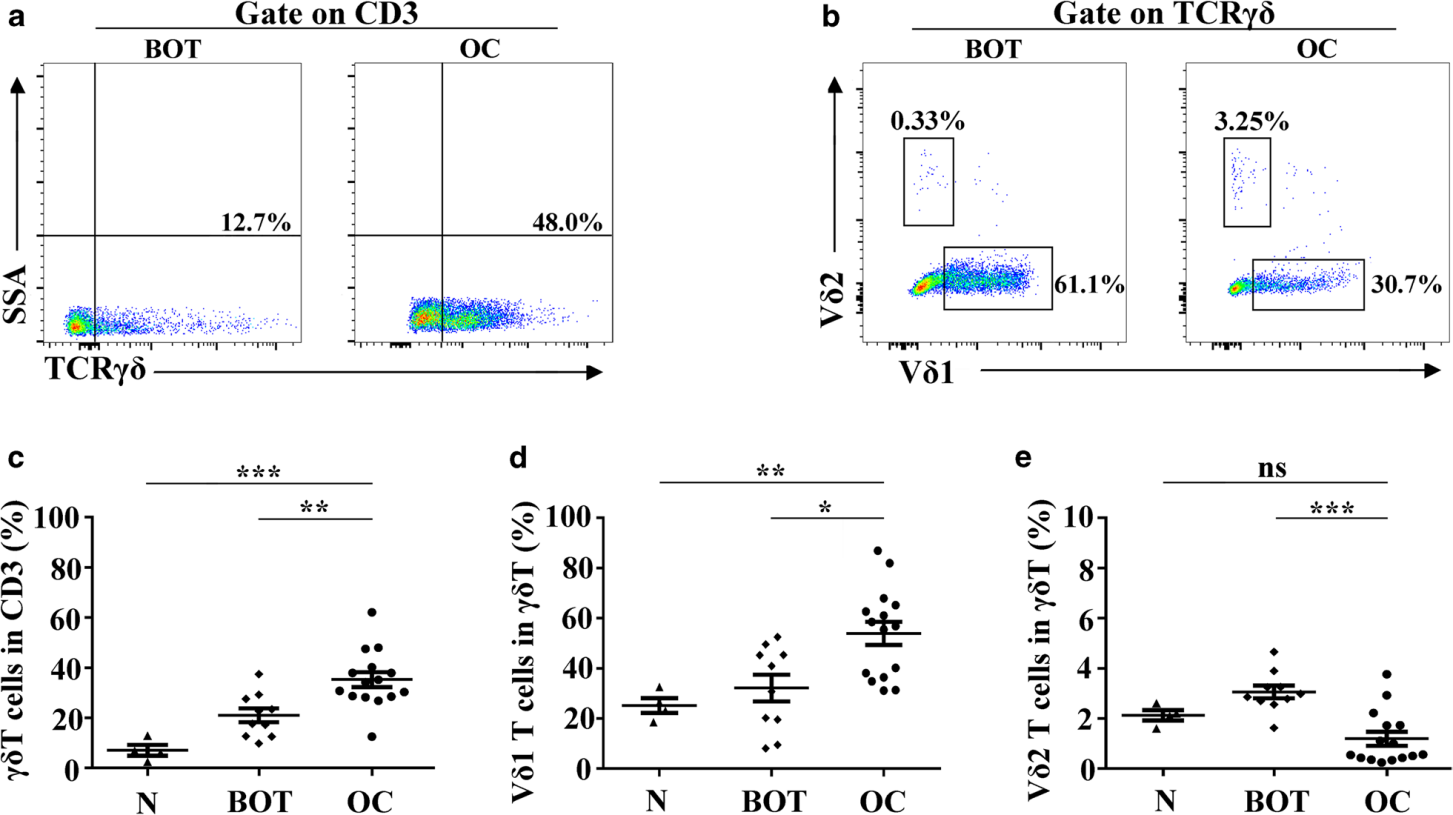
Distribution patterns of γδ T cells in tissues of ovarian cancer. **a**, **b** Representative plots of γδ T cells, Vδ1 T cells and Vδ2 T cells infiltrated in BOT tissues and OC tissues, gated on CD45^+^ CD3^+^ cells. **c**–**e** The proportions of γδ T cells, Vδ1 T cells and Vδ2 T cells infiltrated in normal ovary tissues (n = 4), BOT tissues (n = 10) and OC tissues (n = 15). Data are shown as mean ± SEM, *P < 0.05, **P < 0.01, ***P < 0.001

We next investigated the distinctive distribution patterns of γδ T cells-derived from ovarian tissues. As shown in Fig. 2, the relative percentages of γδ T cells within OC tissues were significantly higher than BOT tissues and N tissues (35.2% ± 11.4% vs. 20.9% ± 8.7%, *P *< 0.01; 35.2% ± 11.4% vs. 7.0% ± 4.3%, *P *< 0.01; Fig. 2a, c). Moreover, Vδ1 T cells, the weakest cohort in PB, exhibited a dominant distribution in ovarian tissues, especially in OC tissues compared with BOT tissues and N tissues (53.9% ± 17.9% vs. 32.1% ± 16.7%, *P *< 0.05; 53.9% ± 17.9% vs. 25.1% ± 5.8%, *P *< 0.01; Fig. 2b, d). Thus, the proportions of Vδ2 T cells were significantly lower in OC tissues than BOT tissues (1.18% ± 1.07% vs. 3.05% ± 0.81%, *P *< 0.01; Fig. 2b, e).

**Fig. 3:**
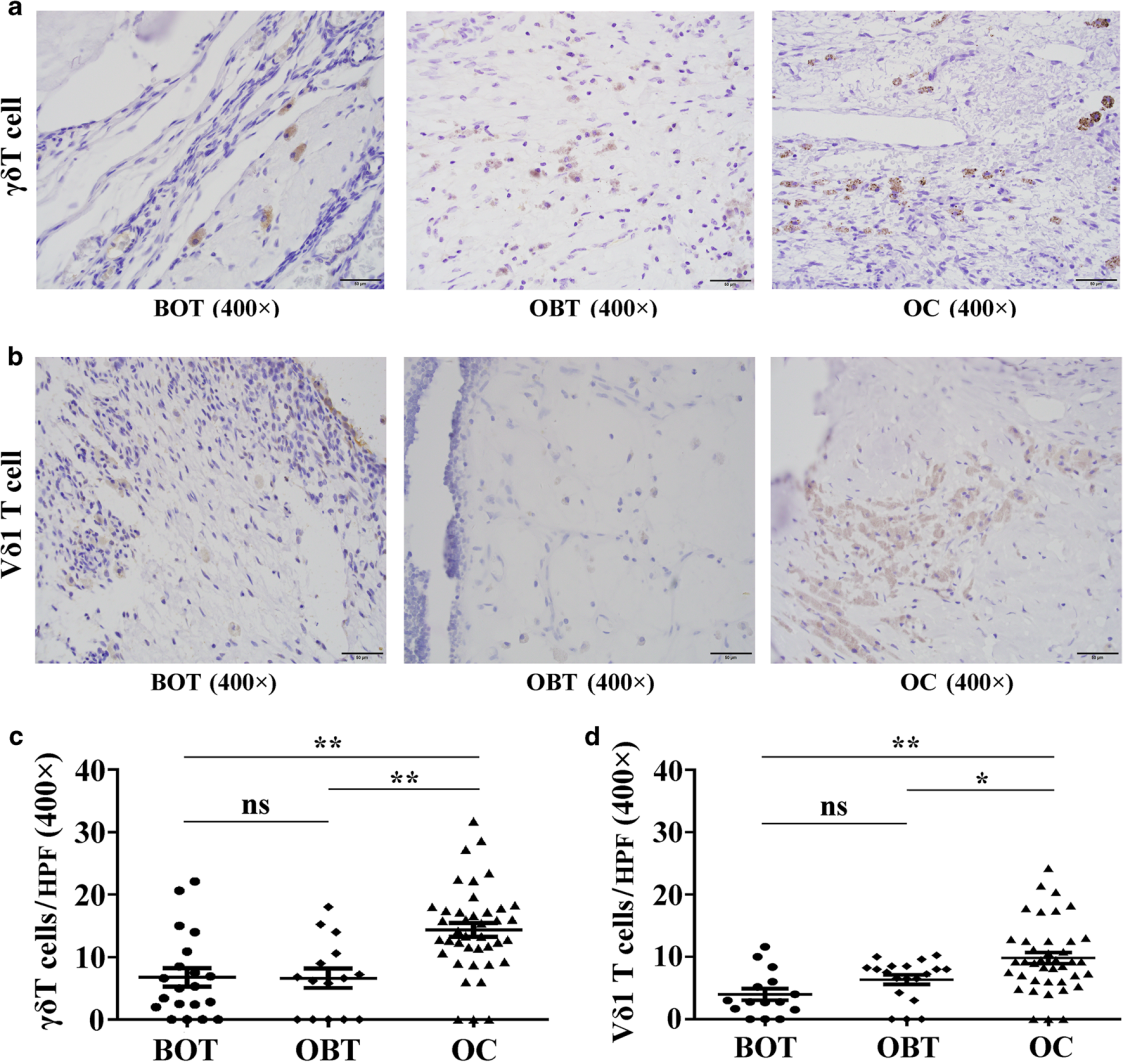
High numbers of γδ T cells and Vδ1 T cells in ovarian cancer tissues by immunohistochemical. **a**, **b** Representative images of γδ T cells and Vδ1 T cells infiltrated in BOT tissues, OBT tissues and OC tissues by IHC. **c**, **d** The numbers of γδ T cells and Vδ1 T cells distributed in OC tissues (n = 40), OBT tissues (n = 20) and BOT tissues (n = 20). Data are shown as mean ± SEM, *P < 0.05, **P < 0.01

We further validated the distribution features of γδ T cells and Vδ1 T cells in BOT, OBT, and OC tissues by IHC as shown in Fig. 3, and found that significantly increased numbers of γδ T cells were displayed in OC tissues, compared with BOT tissues and OBT tissues (*P *< 0.01, *P *< 0.01; Fig. 3a, c). Notably, the higher abundance of γδ T cells exhibited in OC tissues was positively related to advanced clinical FIGO stage, lager tumor size, and lymph node metastasis (*P *< 0.05, *P *< 0.01, *P *< 0.01; Table 1). Additionally, the numbers of Vδ1 T cells were significantly higher in OC tissues than BOT tissues and OBT tissues (*P *< 0.01, *P *< 0.05; Fig. 3b, d), and the numbers of tumor-infiltrating Vδ1 T cells were positively associated with advanced clinical FIGO stage and lymph node metastasis (*P *< 0.05, *P *< 0.05; Table 2). These results indicated that γδ T cells were greatly increased and that Vδ1 T cells were predominant in the tumor microenvironment and may promote the invasiveness and progression of OC.

**Table 1:** Correlation between γδT cells expression and clinicopathological parameters of ovarian cancer

Parameters	Patients (n = 40)	Mean ± SD	*P* value
Ages			
< 50	25	12.28 ± 7.19	0.17
≥ 50	15	15.37 ± 6.52	
Tumor size (cm)			
< 5	7	9.45 ± 6.11	0.04*
≥ 5	33	15.22 ± 6.65	
Histological differentiation			
Serosity	32	14.56 ± 6.98	0.52
Mucinous	8	11.11 ± 2.24	
FIGO stages			
I–II	15	11.45 ± 5.22	0.04*
III–IV	25	15.70 ± 6.82	
Tumor differentiation			
Well	7	13.24 ± 5.47	0.63
Moderate–low	33	14.67 ± 7.34	
Lymphatic metastasis			
Yes	13	16.67 ± 7.15	0.03*
No	27	12.49 ± 5.04	

*P < 0.05

**Table 2:** Correlation between Vδ1 T cells expression and clinicopathological parameters of ovarian cancer

Parameters	Patients (n = 40)	Mean ± SD	*P* value
Ages			
< 50	25	7.76 ± 5.74	0.323
≥ 50	15	9.78 ± 7.24	
Histological differentiation			
Serosity	32	9.34 ± 5.54	0.097
Mucinous	8	5.77 ± 5.54	
FIGO stages			
I–II	15	7.56 ± 4.40	0.04*
III–IV	25	10.87 ± 5.73	
Tumor differentiation			
Well	7	8.14 ± 6.88	0.87
Moderate–low	33	8.56 ± 6.15	
Lymphatic metastasis			
Yes	13	12.00 ± 6.66	0.026*
No	27	7.95 ± 4.05	

*P < 0.05

### IL-17A was highly expressed in γδ T cells of ovarian cancer

**Fig. 4:**
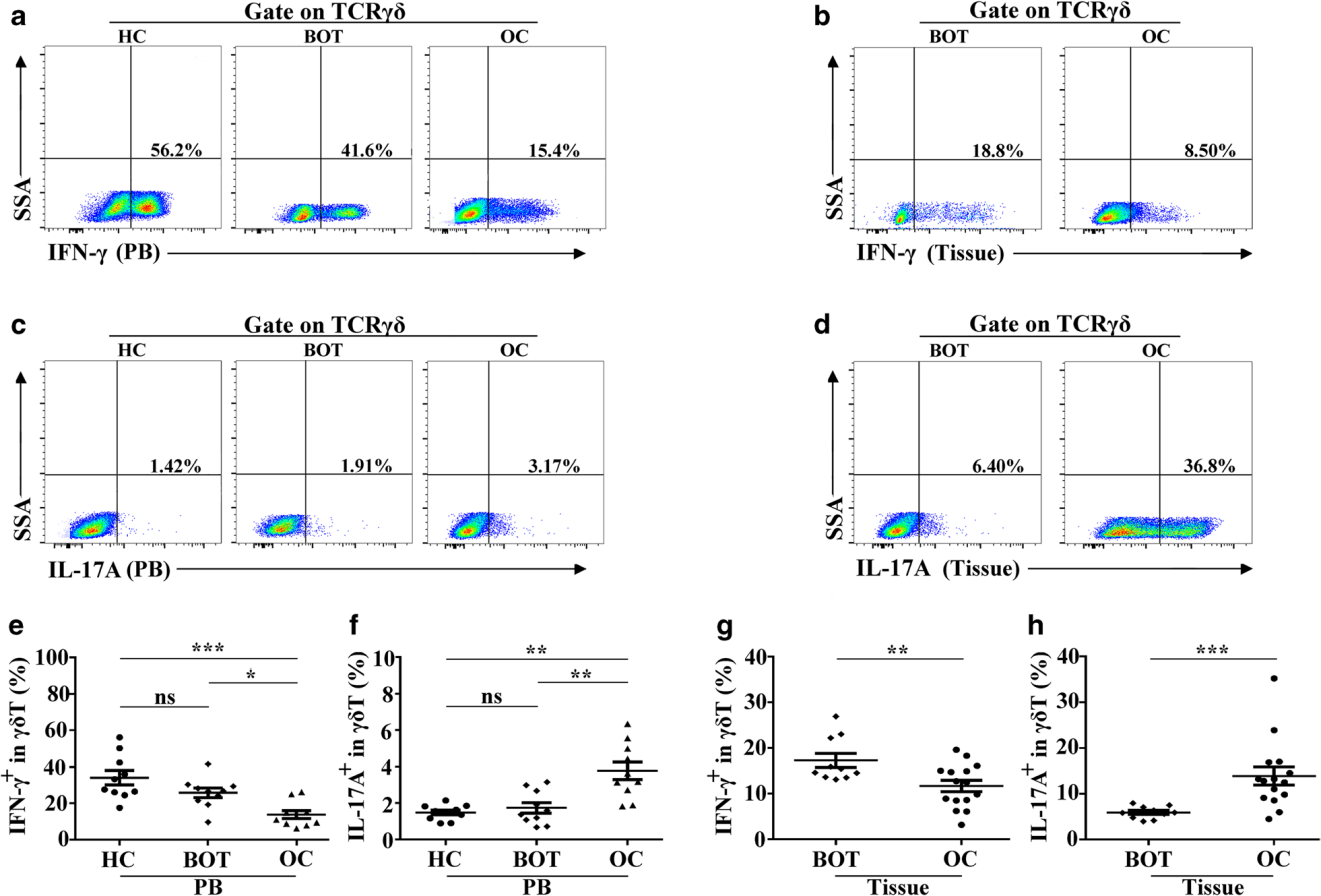
IL-17A highly expressed in γδ T cells of ovarian cancer. **a**, **c** Representative dot pots of IFN-γ and IL-17A secreted by γδ T from peripheral blood in HC, BOT patients and OC patients. **b**, **d** Representative plots of IFN-γ and IL-17A secreted by tumor infiltrated γδ T cells in BOT tissues and OC tissues. **e**, **f** The levels of IFN-γ and IL-17A secreted by γδ T cells in HC (n = 10), BOT patients (n = 10) and OC patients (n = 10). **g**, **h** The levels of IFN-γ and IL-17A in tumor infiltrated γδ T cells of BOT tissues (n = 10) and OC tissues (n = 15). Data are shown as mean ± SEM, *P < 0.05, **P < 0.01, ***P < 0.001

γδ T cells possess the potential to secrete cytokines, and thus we focused on the levels of two intracellular cytokines, IFN-γ and IL-17A; IFN-γ can display cytotoxic effects against infections and tumors, whereas IL-17A has been proven be an important participant in protumor immune responses. As shown in Fig. 4, the levels of IFN-γ secreted from γδ T cells were significantly lower in OC patients compared with BOT patients and HCs in PB (13.7% ± 6.86% vs. 33.98 ± 12.2%, *P *< 0.05; 13.7% ± 6.86% vs. 25.7% ± 8.25%, *P *< 0.001; Fig. 4a, e), but displayed no obvious differences in BOT patients and HCs (25.7% ± 8.25% vs. 33.98 ± 12.2%, *P *> 0.05; Fig. 4a, e). However, the levels of IL-17A showed a completely distinct performance in different cohorts. IL-17A was expressed at higher levels in OC patients compared with BOT patients and HCs (3.7% ± 1.52% vs. 1.73% ± 0.91, *P *< 0.01; 3.7% ± 1.52% vs. 1.48% ± 0.41, *P *< 0.01; Fig. 4c, f) and minimal differences in BOT patients and HCs (1.73% ± 0.91 vs. 1.48% ± 0.41, *P *> 0.05; Fig. 4c, f).

Interestingly, the levels of IFN-γ and IL-17A secreted by tumor-infiltrating γδ T cells revealed similar results. IFN-γ levels were relatively lower in OC tissues than BOT tissues (11.67% ± 4.8% vs. 17.27% ± 4.88%, *P *< 0.01; Fig. 4b, g). However, IL-17A levels were significantly higher in OC tissues than BOT tissues (12.22% ± 4.7% vs. 5.9% ± 1.35, *P *< 0.001; Fig. 4d, h). Furthermore, we compared the levels of IL-17A in PB and tumor tissues of OC patients and found the levels of IL-17A produced by γδ T cells in OC tissues were higher than that in PBof OC patients (*P *< 0.001; Additional file 1: Figure S1). These data suggested that IL-17A was dominantly produced in tumor-infiltrating γδ T cells of OC.

### γδ T cells could be recruited by ovarian cancer tissue supernatants

**Fig. 5:**
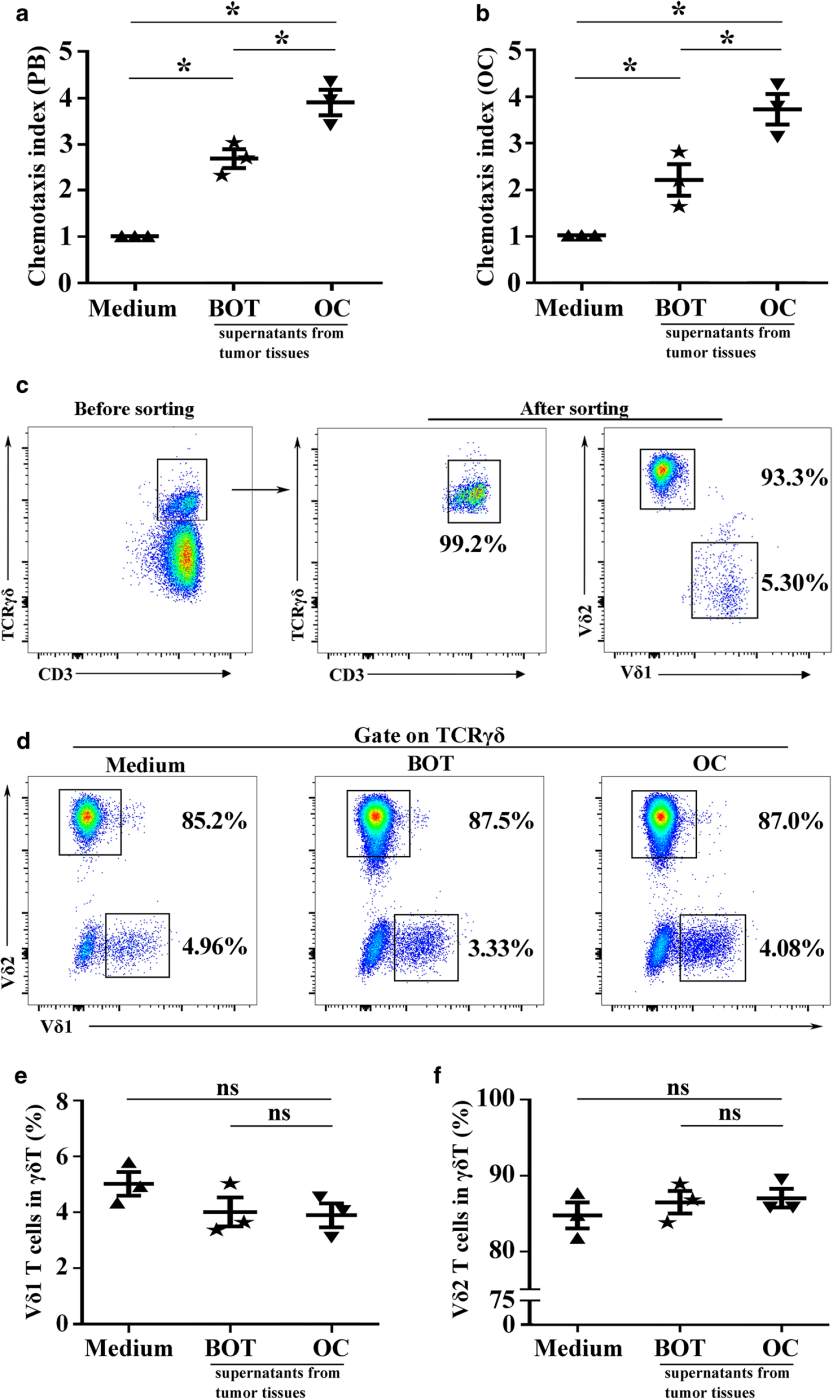
γδ T cells could be recruited by ovarian cancer tissues supernatants. **a**, **b** γδ T cells sorted from peripheral blood (PB) and OC tissues could be superior attracted and migrated by OC tissues supernatants via chemotaxis assay. The data are presented as the mean ± SEM of three independent experiments. *P < 0.05, **P < 0.01. **c** γδ T cells were purified by FACS sorting, and the purities of γδ T cells were greater than 95%. **d**–**f** OC tissues supernatants and BOT tissues supernatants could not convert the Vδ1 T cells and Vδ2 T cells subtype ratio by co-culture experiments. The data are presented as the mean ± SEM of three independent experiments

Due to the high abundance of γδ T cells, both in relative percentages and numbers infiltrated in OC tissues, we continuously investigated whether the OC microenvironment is beneficial for the enrichment of γδ T cells. To test this hypothesis, a chemotaxis assay was executed as shown in Fig. 5. We found that supernatants obtained from fresh OC tissues and BOT tissues caused migrating γδ T cells compared with control medium, but OC tissues supernatants were superior in their ability to attract and migrate γδ T cells from PB and OC tissues compared with BOT tissue supernatants (*P *< 0.05; Fig. 5a, b). Moreover, we also investigated whether supernatants from OC and BOT tissues could convert the subtype of γδ T cells by co-culture experiments in vitro, and found that OC and BOT tissue supernatants could not convert the subtype ratio of Vδ1 T cells and Vδ2 T cells (*P *> 0.05; Fig. 5c–f). Collectively, these data showed that the OC microenvironment could facilitate the accumulation of γδ T cells but could not convert the subtype ratio of Vδ1 T cells and Vδ2 T cells.

### Cytotoxic effects and immunosuppressive activity of γδ T cells in ovarian cancer

**Fig. 6:**
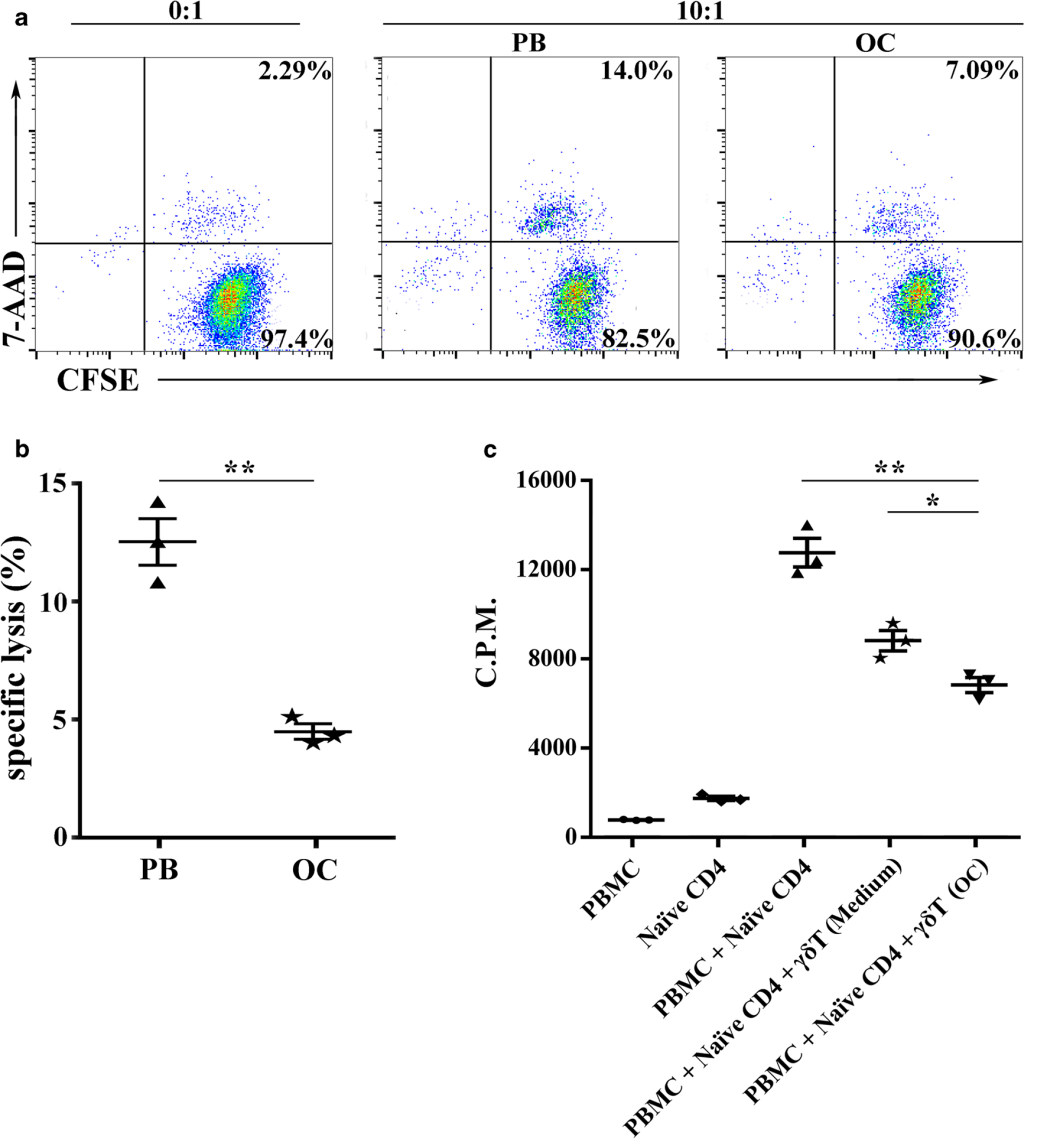
Cytotoxic effects and immunosuppressive activity of γδ T cells in ovarian cancer. **a** Representative charts of the cytotoxic ability of γδ T cells, originated from PB or OC tissues and incubated with CFSE-labeled ovarian cancer cell lines SKOV3 at an effect: target (E:T) ratio of 10:1. **b** γδ T cells from OC tissues killed significantly less ovarian cancer cells SKOV3 than γδ T cells from PB. The data are presented as the mean ± SEM of three independent experiments. **P < 0.01. **c** Suppressive effects of cocultured γδ T cells on naïve CD4^+^ T cells was analyzed in a ratio of 1:1 with stimulation by anti-CD3 and anti-CD28 antibody, irradiated PBMCs were added as APCs. γδ T cells cocultured with OC tissue supernatants could effectively inhibit the proliferative activity of naïve CD4^+^ T cells compared to control medium. C.P.M. is an abbreviation for counts per minute, the data are presented as the mean ± SEM of three independent experiments. *P < 0.05, **P < 0.01

Given the high numbers of γδ T cells infiltrated in OC tissues, we next examined how γδ T cells playa role in the OC microenvironment. We first examined the cytotoxic effects of γδ T cells against human OCSKOV3 cells in vitro. Fresh γδ T cells were sorted from PB of HCs and OC tissues for the cytotoxicity assay. The results revealed that γδ T cells from HCs killed significantly more OC cells than γδ T cells from OC tissues at the same effect: target (E:T) ratio (*P *< 0.01; Fig. 6a, b), which indicated the weakened cytotoxic capacity of γδ T cells in OC tissues.

To this end, we used an in vitro co-culture system to assess the suppressor activity of γδ T cells in naïve CD4^+^ T cell proliferation. γδ T cells were sorted from healthy PB and incubated with OC tissue supernatants or control medium for 5 days. Figure 6c shows the proliferative activity of naïve CD4^+^ T cells with γδ T cells co-cultured with tissue supernatants or control medium. We found that γδ T cells co-cultured with OC tissue supernatants could effectively inhibit the proliferative activity of naïve CD4^+^ T cells compared with control medium in response to anti-CD3 and anti-CD28 antibody stimulation (*P *< 0.05; Fig. 6c). These results indicated that γδ T cells have immunosuppressive activity and may act as ‘inhibitory cells’ in the OC microenvironment to promote the progression of OC.

## Discussion

Investigating the function of TILs in the tumor suppressive microenvironment is essential for the development of effective immunotherapy strategies. In contrast to αβT cells, γδ T cells could recognize and kill various types of tumor cells through MHC-unrestricted lytic activity, suggesting antitumor effects for immunotherapy [11, 12]. Thus, in the current study, we detected the distribution characteristics of γδ T cells and their two main subsets, Vδ1 T cells and Vδ2 T cells. Our results showed that the percentages of γδ T cells, along with Vδ1 T cells and Vδ2 T cells, displayed no obvious differences in the PB of OC and BOT patients and HCs. However, γδ T cells were distributed at higher numbers in OC tissues than BOT tissues and N tissues, and Vδ1 T cells constituted the dominant population, as confirmed by immunohistochemistry. We also demonstrated strong and positive correlations between tumor-infiltrating γδ T cells and Vδ1 T cells with advanced clinicopathological features including clinical FIGO stage, tumor size, and lymph node metastasis. Several studies demonstrated that γδ T cells have critical antitumor activities. It was reported that a higher frequency of γδ T cells in PB was positively correlated with shorter survival rate in advanced renal cell carcinoma patients, while tumor-in filtrating γδ T cells were correlated with a brief disease-free interval in advanced ovarian serous carcinoma [13]. Moreover, an increase in γδ T cells in breast cancer tissue was an independent risk factor for its clinical features. Recently, accumulating evidence showed that different γδ T cells subsets have different immunity effects, and particularly highlighted the immunosuppression role of Vδ1 T cells in epithelial-derived tumors including human rectal cancer and breast cancer, where their presence is negatively correlated with clinical outcome [14, 15]. Therefore, our results suggested that tumor-infiltrating γδ T cells and Vδ1 T cells may perform critical roles in promoting tumor invasiveness and progression of OC [16, 17].

After decades of research, inflammation was designated as a hallmark of cancer [18]. However, it is suggested that endometriosis might increase the risk of invasive epithelial OC via the formation of a chronic inflammatory milieu in the ovary [19, 20]. Various cytokines and chemokines derived from tumor milieu can activate inflammation-related signaling molecules and recruit immunosuppressive cells, leading to immunosuppression and prompting tumor angiogenesis and progression [21, 22]. IFN-γ is an active conventional cytokine in anti-tumor immunity, while IL-17A is conversely considered to promote immunosuppression and participate in tumor immune escape. It was demonstrated that IL-17A was over expressed in cervical cancer, breast cancer, hepatocellular carcinoma, non-small cell lung cancer, and pancreatic cancer [23–25]. Therefore, we investigated the levels of IFN-γ and IL-17A secreted by γδ T cells in PB and tissues, and found higher levels of IL-17Ain both PB and tissues of OC patients, but lower levels of IFN-γ secreted by γδ T cells, while IL-17A was inclined to infiltrate the tumor milieu of OC. It was previously reported IL-17A-producing γδ T cells are capable of inducing tumor angiogenesis in human gallbladder cancer, and promote OC growth via mobilization of protumor small peritoneal macrophages in a mouse model [26]. A study of tumor-infiltrating γδ T cells revealed that IL-17A could polarize inflammatory macrophages and recruit MDSCs to the tumor site of colorectal cancer, and also recruit neutrophils to tumor sites to boost breast cancer metastasis, leading to an immunosuppressive microenvironment [27]. Thus, our results suggested that IL-17A was dominantly produced in tumor-infiltrating γδ T cells of OC, which may indicate the suppressive roles that IL-17A plays in the OC microenvironment.

Based on our data showing that increased numbers of γδ T cells were present in ovarian tumor tissues but not in normal ovarian tissues and were correlated with clinicopathological features, we next evaluated why γδ T cells accumulate in OC tissue and what role they play in the tumor milieu of OC. It was reported that IP-10 secreted by breast cancer cells could be responsible for migrating and trafficking γδ T cells to tumor sites [10, 15]. Thus, one critical mechanism responsible for the accumulation of γδ T cells within the ovarian tumor microenvironment is the preferential recruitment of these cells. Importantly, our current research strongly implies that supernatants from the OC microenvironment could promote the accumulation of γδ T cells by attracting and recruiting γδ T cells to the tumor microenvironment, although this did not result in the conversion of Vδ1 T cells and Vδ2 T cells in this study. Thus, our future studies will focus on the mechanisms that contribute to the attraction and conversion of γδ T cells in the OC microenvironment.

Most recent reports suggest that γδ T cells can facilitate tumor progression. γδ T cells infiltrated in pancreatic cancer tissues can restrain CD4^+^ T cell and CD8^+^ T cell activation by expressing high levels of the immune check point proteins PD-L1 and Galectin-9, thereby negating adaptive anti-tumor immunity [17]. Breast cancer-infiltrating γδ T cells block the maturation of dendritic cells and suppress the immune response of αβT cells [28], while γδ T cells of colorectal cancer meditate the elicitation of tumor-related inflammation and help to establish an immunosuppression network. In our study, we established an in vitro cytotoxicity assay system and proliferation assay to explore the function of the numerous γδ T cells in the OC microenvironment, and found that γδ T cells from OC tissues have a weakened ability to kill OC cells. Moreover, γδ T cells co-cultured with OC tissue supernatants could enhance the suppression of naïve CD4^+^ T cell proliferation. Several views may explain the complex interaction between γδ T cells and OC cells. γδ T cells can limit the proliferation of ovarian tumor cells by downregulating apoptosis and cell cycle-related molecules, while γδ T cells combined with induced ATM/ATR signaling could enhance the cytotoxicity to OC cells; moreover, ovarian tumor cells could escape γδ T cell-mediated recognition by upregulating pERK1/2 and downregulating surface MICA levels [29, 30]. Collectively, these data suggested the impaired anti-tumor cytotoxic effects and enhanced immunosuppressive function of γδ T cells in OC, which indicated that γδ T cells may act as ‘inhibitory cells’ for creating an immunosuppressive microenvironment to limit antitumor immunity, evade immune surveillance, and facilitate the progression of OC.

## Conclusions

Taken together, our study indicates that theOC microenvironment might mobilize and recruit γδ T cells, which secrete large amounts of the inflammatory cytokine IL-17A, have weakened cytotoxic effects, and enhance immunosuppressive activity to establish a potent immunosuppressive microenvironment for promoting tumor progression and immune evasion. In conclusion, γδ T cells might be a prognostic factor of OC, provide a good target for immunotherapy, and enable optimization of immunotherapy.

## Additional file

**Additional file 1: Figure S1.** Related to Fig. 4. IL-17A highly expressed in γδ T cells of ovarian cancer. The levels of IL-17A secreted by tumor infiltrated γδ T cells of OC tissues (n = 15) was higher than that of peripheral blood (n = 10). Data are shown as mean ± SEM, ***P < 0.001.

## Data Availability

The datasets used and analysed during the current study are available from the corresponding author on reasonable request.

## Abbreviations

OC: ovarian cancer
BOT: benign ovarian tumor
OBT: borderline ovarian tumor
HC: healthy controls
PBMC: peripheral blood mononuclear cells
IHC: immunohistochemistry
FACS: fluorescence activated cell sorting
