# Supplementary figures and images for: Distribution and functions of γδ T cells infiltrated in the ovarian cancer microenvironment
Source: J Transl Med. 2019 May 7;17:144. doi: 10.1186/s12967-019-1897-0 (PMC6505080; doi:10.1186/s12967-019-1897-0)

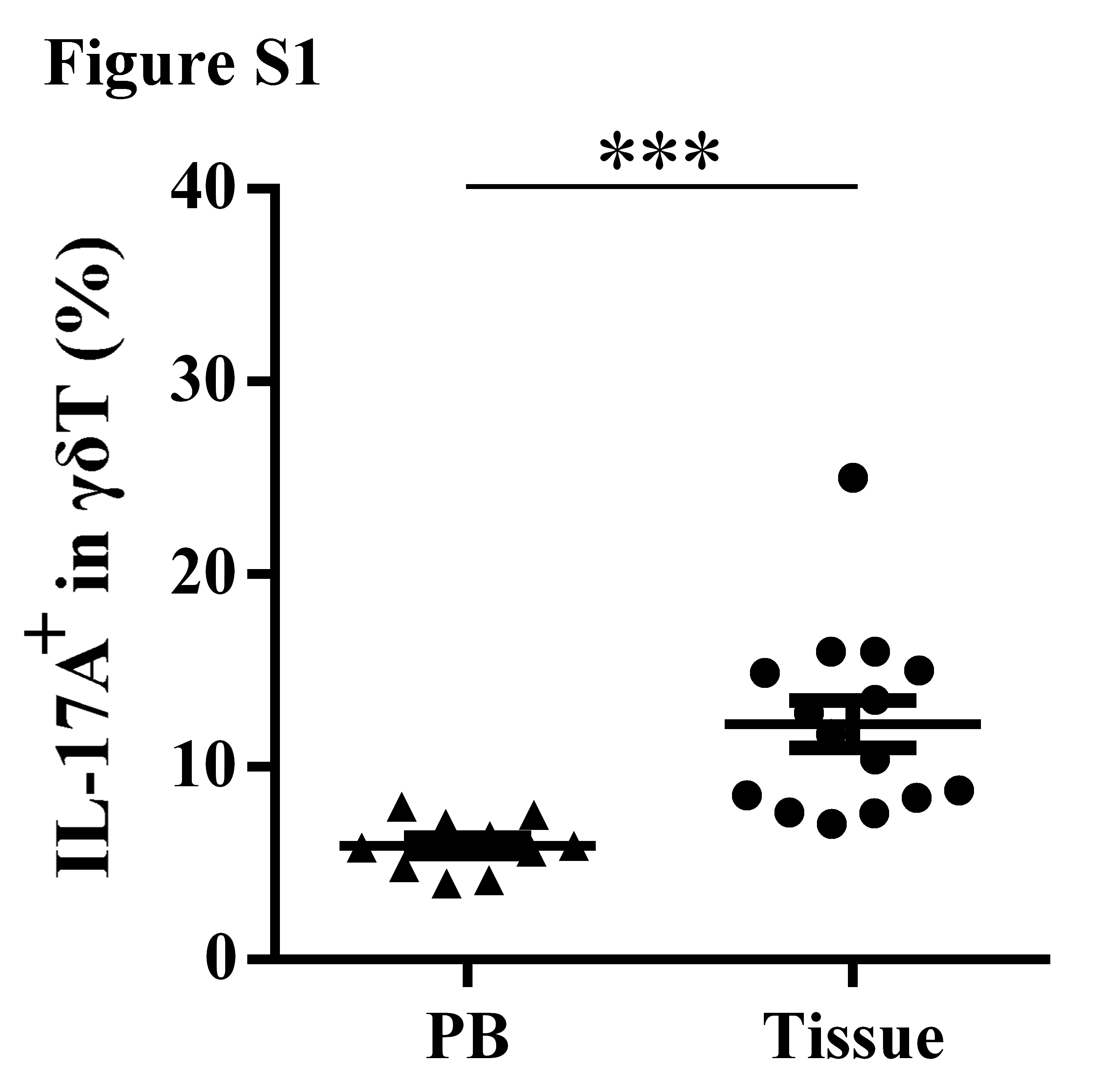

Supplement: Supplementary file 1 — Additional file 1: Figure S1. Related to Fig. 4. IL-17A highly expressed in γδ T cells of ovarian cancer. The levels of IL-17A secreted by tumor infiltrated γδ T cells of OC tissues (n = 15) was higher than that of peripheral blood (n = 10). Data are shown as mean ± SEM, ***P < 0.001. [file 12967_2019_1897_MOESM1_ESM.tif]
